# Supplementary material for: The utilisation of public and private health care among Australian women with diabetes: Findings from the 45 and Up Study
Source: PLoS One. 2021 Aug 12;16(8):e0255573. doi: 10.1371/journal.pone.0255573 (PMC8360374; doi:10.1371/journal.pone.0255573)
Supplement: Attachment — Submitted filename: Response to Reviewers_25.05.2021.docx [file pone.0255573.s001.docx]

**Editor’s comment**: Thank you for providing additional details regarding the restrictions on data sharing. Before we proceed, please confirm that the authors had no special access to the data, and that all qualified researchers can request access to the data in the same way the authors obtained it.

- If these are true, please also confirm the following Data Availability Statement is accurate, and we will update it on your behalf:

"Data cannot be shared publicly as they are owned by the 45 and Up study. However, anyone wishing to request access can do so through the 45 and Up study. Requests can be submitted via email to [45andUp@saxinstitute.org.au](mailto:45andUp@saxinstitute.org.au) for researchers who meet the criteria for access to confidential data."

**Response**: The data was originally only available to the research team but is now available to any researchers who meet the criteria for access to confidential data. As such, the proposed Data Availability Statement is accurate and can be include in the document.

Editor’s comment: Please ensure that your related previously published article is referenced in the manuscript.

Response: Thank you for the opportunity to add this detail to the manuscript. The methods section has now been amended as follows (see underlined):

“*The authors obtained data from a sub-study of the Sax Institute’s 45 and Up Study, described in detail elsewhere (21). The baseline questionnaire collected information from 266,848 men and women who reside in the State of New South Wales, Australia and are aged 45 years and above. To establish the 45 and Up Study, individuals meeting this criteria were randomly selected from the Medicare Australia database which provides virtually complete coverage of the general population. Participants entered the study by completing a baseline postal questionnaire between January 2006 and December 2009 and consenting to have their health followed over time. The study reported here is one of a set of sub-studies of women from this cohort who had previously indicated that they had been diagnosed with one of five chronic illnesses: asthma, arthritis, depression, diabetes and osteoporosis). Details of those associated sub-studies have been published elsewhere (e.g. 22-27). For this sub-study, the research team mailed a questionnaire between August and November, 2016 to 800 women who reported having been diagnosed with diabetes by a doctor; 392 returned the questionnaire, representing a response rate of 49.0%*.”

REFS:

22. Adams, J., Steel, A., Frawley, J., Leach, M., McIntyre, E., Broom, A., & Sibbritt, D. (2020). The health care utilization and out-of-pocket expenditure associated with asthma amongst a sample of Australian women aged over 45 years: analysis from the ‘45 and up’study. *Journal of Asthma*, 1-9.

23. Lauche, R., Sibbritt, D., Adams, J., & Cramer, H. (2019). Characteristics of yoga and meditation users among older Australian women–results from the 45 and up study. *Complementary therapies in clinical practice*, *35*, 219-223.

24. McIntyre, E., Lauche, R., Frawley, J., Sibbritt, D., Reddy, P., & Adams, J. (2019). Physical activity and depression symptoms in women with chronic illness and the mediating role of health-related quality of life. *Journal of affective disorders*, *252*, 294-299.

25. Adams, J., Prior, J., Sibbritt, D., Connon, I., Dunston, R., McIntyre, E., & Lauche, R. (2018). The use of self-care practices and products by women with chronic illness: a case study of older women with osteoarthritis and osteoporosis. In *Women’s Health and Complementary and Integrative Medicine* (pp. 77-93). Routledge.

26. Adams, J., McIntyre, E., Frawley, J., Lauche, R., Broom, A., & Sibbritt, D. (2019). Formal and informal healthcare behaviours of women with chronic illness: A cross‐sectional analysis of 1925 women. *International journal of clinical practice*, *73*(4), e13343.

27. Meredith, S., Frawley, J., Sibbritt, D., & Adams, J. (2020). Risk factors for developing comorbid sleeping problems: results of a survey of 1,925 women over 50 with a chronic health condition. *Journal of aging and health*, *32*(5-6), 472-480.
